# Memantine potentiates cytarabine-induced cell death of acute leukemia correlating with inhibition of K_v_1.3 potassium channels, AKT and ERK1/2 signaling

**DOI:** 10.1186/s12964-018-0317-z

**Published:** 2019-01-16

**Authors:** Theresa Lowinus, Florian H. Heidel, Tanima Bose, Subbaiah Chary Nimmagadda, Tina Schnöder, Clemens Cammann, Ingo Schmitz, Ulrike Seifert, Thomas Fischer, Burkhart Schraven, Ursula Bommhardt

**Affiliations:** 1 Institute of Molecular and Clinical Immunology, Health Campus Immunology, Infectiology and Inflammation (GC-I3), Otto-von-Guericke-University Magdeburg, Leipziger Str. 44, 39120 Magdeburg, Germany; 2 Department of Hematology and Oncology, GC-I3, Otto-von-Guericke-University Magdeburg, Magdeburg, Germany; 3 Leibniz Institute on Aging, Fritz-Lipmann Institute, Jena, Germany; 4 Innere Medizin II, Universitätsklinikum Jena, Jena, Germany; 5 Leibniz Institute of Neurobiology, Magdeburg, Germany; 6 Friedrich Loeffler Institute for Medical Microbiology, University Medicine Greifswald, Greifswald, Germany; 7 Systems-Oriented Immunology and Inflammation Research Group, Helmholtz Centre for Infection Research, Braunschweig, Germany; 8 Department of Immune Control, Helmholtz Centre for Infection Research, Braunschweig, Germany; 9 Present address: Department of Hematology, Oncology, and Stem Cell Transplantation, Faculty of Medicine, Freiburg University Medical Center, Freiburg, Germany; 10 Present address: Institute for Clinical Neuroimmunology, Ludwigs-Maximilians-University, Munich, Germany

**Keywords:** Memantine, Acute leukemia, Cell death, Cytarabine, Signaling

## Abstract

**Background:**

Treatment of acute leukemia is challenging and long-lasting remissions are difficult to induce. Innovative therapy approaches aim to complement standard chemotherapy to improve drug efficacy and decrease toxicity. Promising new therapeutic targets in cancer therapy include voltage-gated K_v_1.3 potassium channels, but their role in acute leukemia is unclear. We reported that K_v_1.3 channels of lymphocytes are blocked by memantine, which is known as an antagonist of neuronal N-methyl-D-aspartate type glutamate receptors and clinically applied in therapy of advanced Alzheimer disease. Here we evaluated whether pharmacological targeting of K_v_1.3 channels by memantine promotes cell death of acute leukemia cells induced by chemotherapeutic cytarabine.

**Methods:**

We analyzed acute lymphoid (Jurkat, CEM) and myeloid (HL-60, Molm-13, OCI-AML-3) leukemia cell lines and patients’ acute leukemic blasts after treatment with either drug alone or the combination of cytarabine and memantine. Patch-clamp analysis was performed to evaluate inhibition of K_v_1.3 channels and membrane depolarization by memantine. Cell death was determined with propidium iodide, Annexin V and SYTOX staining and cytochrome C release assay. Molecular effects of memantine co-treatment on activation of Caspases, AKT, ERK1/2, and JNK signaling were analysed by Western blot. K_v_1.3 channel expression in Jurkat cells was downregulated by shRNA.

**Results:**

Our study demonstrates that memantine inhibits K_v_1.3 channels of acute leukemia cells and in combination with cytarabine potentiates cell death of acute lymphoid and myeloid leukemia cell lines as well as primary leukemic blasts from acute leukemia patients. At molecular level, memantine co-application fosters concurrent inhibition of AKT, S6 and ERK1/2 and reinforces nuclear down-regulation of MYC, a common target of AKT and ERK1/2 signaling. In addition, it augments mitochondrial dysfunction resulting in enhanced cytochrome C release and activation of Caspase-9 and Caspase-3 leading to amplified apoptosis.

**Conclusions:**

Our study underlines inhibition of K_v_1.3 channels as a therapeutic strategy in acute leukemia and proposes co-treatment with memantine, a licensed and safe drug, as a potential approach to promote cytarabine-based cell death of various subtypes of acute leukemia.

**Electronic supplementary material:**

The online version of this article (10.1186/s12964-018-0317-z) contains supplementary material, which is available to authorized users.

## Background

Therapy of acute leukemia demands high dose chemotherapy and often stem cell transplantation, which is not feasible in elderly leukemia patients due to severe toxic side effects. For those patients, prognosis remains poor and even palliative treatment options are limited. In addition, older patients show a more heterogeneous clinical biology with induction of aberrant signaling pathways. Therefore, to complement standard chemotherapy and to improve drug efficacy, multi-targeting approaches with potential drug combinations are being investigated [1, 2]. The PI3K-AKT-mTOR and MAPK/ERK1/2 pathways are major signaling cascades deregulated in acute leukemia [3–7] and contribute to an aggressive phenotype and enhanced chemo-resistance. An important target of ERK1/2 [8] and AKT [9] signaling is the transcription factor MYC, which is involved in cell growth, proliferation and apoptosis [10]. Since MYC is a central oncogene inducing leukemic transformation in T cell acute lymphoid leukemia (T-ALL) and acute myeloid leukemia (AML) [11, 12], influencing AKT, ERK1/2 and MYC signaling may enhance the efficacy of chemotherapy in ALL and AML [13–20].

In search of novel treatment options, voltage-gated K_v_1.3 potassium channels have become promising drug targets. K_v_1.3 channels localize in the plasma membrane and participate in controlling the membrane potential, proliferation and effector function of lymphocytes [21]. In addition, inactivation of K_v_1.3 channels expressed in the inner mitochondrial membrane [22] induces intrinsic apoptosis of lymphocytes via mitochondrial cytochrome C (CytC) release and production of reactive oxygen species (ROS) [23–25]. K_v_1.3 channels on lymphoid and myeloid leukemia cells have been proposed as diagnostic biomarkers [26, 27] and druggable targets for therapy in chronic lymphocytic leukemia [25, 28], lymphoma [29], and solid cancers [30–34]. However, the role of K_v_1.3 channels in acute leukemia is unclear and there are no licensed drugs for specific inhibition of K_v_1.3 channels in cancer treatment.

Memantine (3,5-Dimethyltricyclo [3.3.1.1] decanamin) is a registered drug known to inhibit N-methyl-D-aspartate type glutamate receptors (NMDARs) in neurons and has been used for many years in the treatment of moderate-to-severe Alzheimer disease [35, 36]. We reported that in lymphocytes memantine blocks K_v_1.3 channel activity and diminishes lymphocyte effector function [37, 38]. Furthermore, standard doses of memantine applied in Alzheimer therapy inhibit K_v_1.3 channels on patients’ peripheral blood T cells and alter T cell reactivity in vivo [39]. Thus, we asked whether pharmacological inhibition of K_v_1.3 channels by memantine could be an option to enhance cell death of acute leukemia cells induced by the chemotherapeutic drug cytarabine (AraC). Analyzing acute lymphoid and myeloid leukemia cell lines and patients’ acute leukemic blasts, our data highlight the importance of K_v_1.3 channels for the survival of acute leukemia cells and provide initial evidence that memantine and cytarabine co-treatment may be a potential therapeutic strategy to enhance the efficacy in acute leukemia treatment.

## Methods

### Cell culture and determination of cell death

Jurkat (JE6–1), F9, JMR [40], A3, C8 (I.9.2) [41], CEM, HL-60, Molm-13, OCI-AML-3 cells, primary cells from healthy donors, and primary leukemic cells were cultured in RPMI 1640 medium (Biochrom AG, Berlin, Germany) supplemented with 10% fetal calf serum. JE6–1 and CEM cells were bought from ATCC and AML cell lines from DSMZ or ATCC. Cell lines were tested for mycoplasma with PCR or Mycoplasma Detection Kit from Lonza (Basle, Switzerland). Peripheral blood was obtained from healthy donors and a newly diagnosed T-ALL patient, and bone marrow (BM) samples of AML patients were obtained from the Tumor Bank of the Medical Faculty Magdeburg. Peripheral blood mononuclear cells (PBMC) were isolated with Biocoll (Biochrom AG) and CD3^+^ T cells with Pan-T-Cell-Isolation Kit-2 (Miltenyi, Bergisch-Gladbach, Germany). In each experiment, cell lines or primary cells were cultured without drug, with memantine (Tocris Biosciences, Bristol, Great Britain), AraC (Department of Pharmacy, Medical Faculty Magdeburg), and a combination of memantine plus AraC. AraC concentrations were titrated for each ALL and AML cell line to cover 10–90% cell death. Cell death (with gating on all cells or specific subpopulations when indicated) was determined with SYTOX-Pacific Blue™ (Molecular Probes, Thermo Fisher Scientific, Darmstadt, Germany) ± Annexin V-FITC (BD Pharmingen, Heidelberg, Germany), propidium iodide (PI) (Sigma-Aldrich, St. Louis, USA) or LIVE/DEAD™ Fixable Aqua Dead Cell Stain Kit (Invitrogen, Thermo Fisher Scientific). Percentage of T-ALL cells in sub-G_0/1_-phase of cell cycle was determined with PI. K_v_1.3 surface expression was analysed with K_v_1.3-FITC antibodies (Alomone, Jerusalem, Israel; Sigma-Aldrich) using IgG isotype-FITC (BD Pharmingen) or unstained cells as controls. CEM and AML cell lines were incubated with human FcR block (Miltenyi) before staining with K_v_1.3 antibodies. BM samples from AML patients were thawed and cultured for 3 or 24 h. Cells were stained with LIVE/DEAD Aqua Blue to discriminate live cells, incubated with human FcR block and then labeled with CD117 (104D2, BioLegend) and K_v_1.3 antibodies. K_v_1.3 expression is shown for viable CD117^+^ leukemic blasts. Flow cytometry was performed with a FACSFortessa™ (BD Bioscience, Mountain View, USA).

### Determination of ATP content and cytochrome c (CytC) release

Intracellular ATP content, as a measure of viable cells, was determined with CellTiter-Glo® Luminescent Cell Viability Assay (Promega, Mannheim, Germany) according to the manufacturer’s protocol. Mitochondrial CytC release was determined as described [42]. In brief, plasma membranes of cultured cells were permeabilized with the mild detergent saponin. Cells were washed to remove cytosolic CytC released from mitochondria during apoptosis. Then the outer mitochondrial membrane was permeabilized with digitonin (50 μg/ml) in 50 μM EDTA, 100 mM KCl for 5 min on ice. Cells were fixed in 4% paraformaldehyde for 20 min, washed and incubated for 1 h in blocking buffer (3% BSA, 0.05% saponin in PBS). Cells were incubated overnight with CytC-AlexaFluor 488 monoclonal antibodies (6H2.B4, BD Pharmingen) and analyzed by flow cytometry. Cells having released mitochondrial CytC, i.e. apoptotic cells, had a lower fluorescence signal.

### Proliferation assay

DNA synthesis was determined in triplicates by ^3^[H]-Thymidine incorporation (0.2 μCi/ well, MP Biomedicals, Heidelberg, Germany) at day 3 for 16 h.

### Electrophysiology

Patch-clamp experiments were performed as described [37]. K_v_1.3 currents were measured every 30 s with depolarizing voltage steps up to + 60 mV from a holding potential of − 80 mV. Sampling rate was 50 kHz during the measurement of K_v_1.3 currents. Number of K_v_1.3 channels per cell was determined by dividing the whole-cell K_v_1.3 conductance by the single-channel conductance value (K_v_1.3:12 pS) [43, 44]. For membrane potential experiments, JE6–1 cells were recorded in the current clamp mode with zero pA holding current immediately after establishment of the whole-cell configuration. Memantine was kept in a constant concentration during recording in the fixed holding potential (− 80 mV) and the amplitude of the current was measured to determine membrane depolarization. KCl treatment served as a positive control for cell integrity.

### Immunoblotting

Cells were cultured without drug, with memantine, AraC (60 nM for Jurkat, 250 nM for Molm-13), and memantine plus AraC. Cytoplasmic and nuclear protein extracts were prepared as described [37]. Antibodies used in Western blots were: p-AKT(S473), AKT, pS6(S240/244), p-ERK1/2(T202/Y204), ERK1/2, pJNK1/2(Thr183/Tyr185), c-JUN (60A8), human Caspase-9, active Caspase-3 (5A1E, only detects cleaved active Caspase-3), Caspase-8 (all Cell Signaling, Leiden, The Netherlands), c-MYC (9E10, BD Pharmingen), β-Actin (AC-74, Sigma-Aldrich), Lamin B (sc-6217, Santa Cruz Biotechnology Europe). Primary antibodies were detected with species-specific secondary antibodies (Dianova, Hamburg, Germany) and chemiluminescence. Nitrocellulose membranes were reprobed for several proteins.

### Lentiviral transduction

Lentiviral transduction was performed as described [45]. Lentivirus was generated in 293 T cells by transfection of pLKO.1-K_v_1.3 shRNA or pLKO.1-scrambled (scr) shRNA (Sigma-Aldrich). JE6–1 cells were infected twice with lentivirus. Puromycin was kept at 0.25 μM for 4 days. RNA was extracted at day 3–4 post infection using Trizol® (Invitrogen, Thermo Fisher Scientific) and reverse transcribed (Quiagen, Düsseldorf, Germany). Knockdown was confirmed by PCR: forward K_v_1.3: 5′-GGT CAT CAA CAT CTC CGG CGT GCG CT-3′ and reverse K_v_1.3: 5′-AGG GCC GCT CCT CCT CCC GC-3′ (Apara Bioscience, Denzlingen, Germany), SYBR Green II (Thermo Fisher Scientific) and the CFX96 Real-Time System (BIO-RAD, Munich, Germany).

### Statistical analysis

Statistics were performed with Cell Quest Pro software (BD Bioscience), HEKA FitMaster v2x53 and IgorPro for patch-clamp transient currents and GraphPad Prism for analysis of dose-response curves and Student’s *t*-test, with P* < 0.05, P** < 0.01, and P*** < 0.001. The combination index (CI) and dose reduction index (DRI) were determined with CompuSyn1.0 software using the Chou-Talalay method [46]. For primary AML cells, the coefficient of drug interaction (CDI) was calculated to assess the synergistic inhibitory effect of the drug combination (CDI = AB/(A × B)) [47].

## Results

### Inactivation of K_v_1.3 channels promotes cell death of acute leukemia T cells

**Fig. 1:**
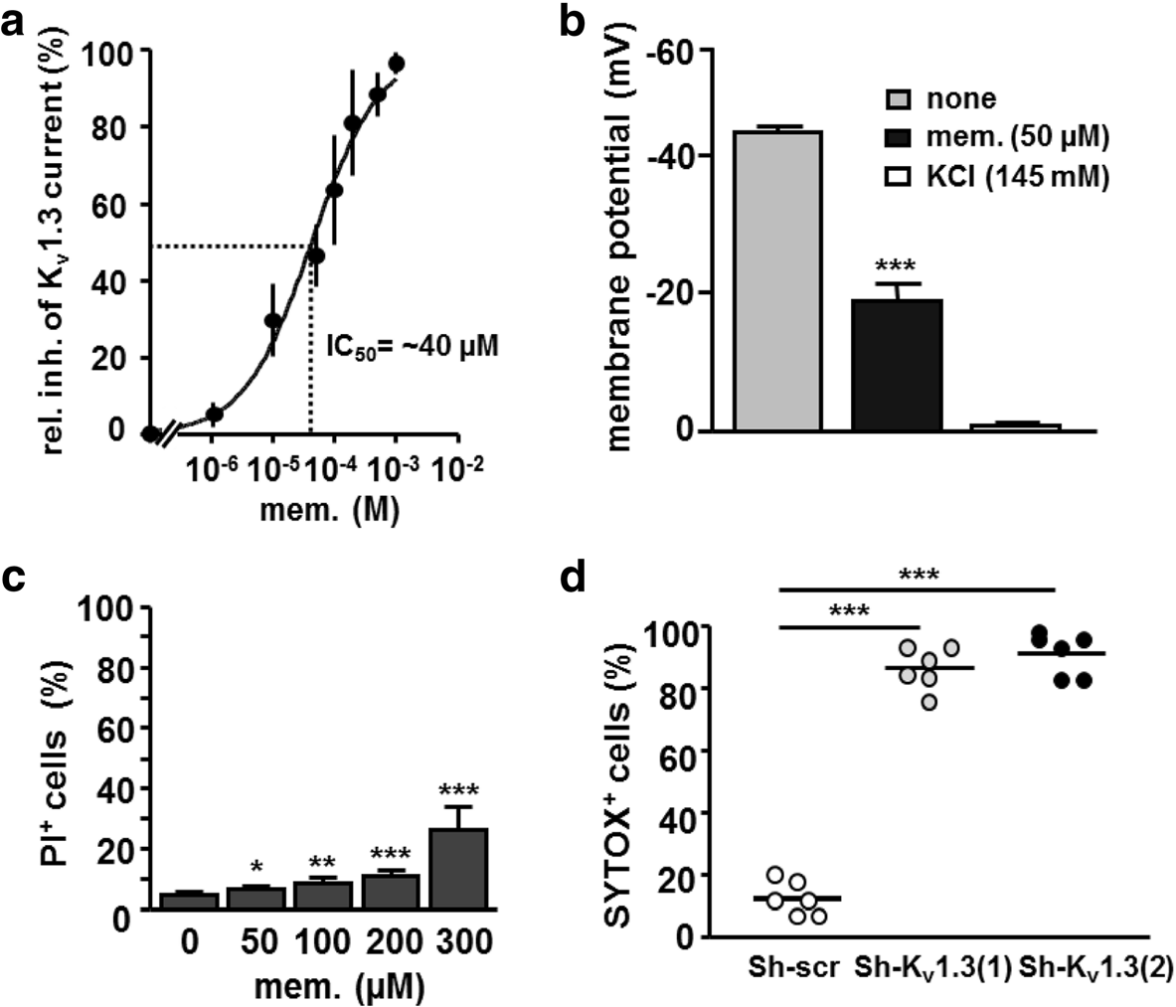
Pharmacological inhibition by memantine and genetic downregulation of K_v_1.3 channels promote cell death of acute lymphoblastic leukemia cells. **a** The graph shows the dose response relationship for memantine of isolated K_v_1.3 currents from Jurkat (JE6–1) cells. Each data point represents the mean ± SD of 5–7 cells from *n* = 3 independent experiments. **b** The membrane potential of untreated and memantine-treated Jurkat cells was determined by current clamp. KCl treatment served as a positive control; the mean + SD was calculated from 5 to 6 cells of *n* = 3 independent experiments. **c** Jurkat cells were treated with the indicated concentrations of memantine for 72 h and cell death was determined with propidium iodide (PI) staining and flow cytometry. Data gives the mean + SD percentage of PI^+^ cells from *n* = 5 independent experiments. **d** Jurkat cells were infected with lentivirus harboring shRNA against K_v_1.3 channels (Sh-K_v_1.3 (1) and Sh-K_v_1.3 (2)) or scrambled sequence (Sh-scr). SYTOX staining was performed at day 7. Percentage and mean of SYTOX^+^ cells is shown for *n* = 6 independent experiments

K_v_1.3 channels play a key role in setting the resting potential, proliferation and apoptosis of T cells and are expressed in healthy human T cells and the T-lymphoblastic leukemia cell line Jurkat [21, 48, 49] (Additional file 1: Figure S1a). Given that specific K_v_1.3 channel blockers for clinical therapy are not licensed, we used memantine, an approved drug shown to block K_v_1.3 channels in vivo [39], and analyzed its effect on Jurkat cells. In voltage-clamp recordings, memantine blocked K_v_1.3 channel currents of Jurkat cells in a dose-dependent manner with an IC_50_ value of ~ 40 μM (Fig. 1a). Memantine depolarized the membrane potential (Fig. 1b) and induced substantial cell death of Jurkat cells at concentrations above 200 μM (Fig. 1c). To support a role of K_v_1.3 channels in survival of acute leukemia cells, we knocked down K_v_1.3 channels in Jurkat cells. Lentiviral transduction of Jurkat cells resulted in a 40–50% reduction of K_v_1.3 mRNA (Additional file 1: Figure S1b). Partial knockdown of K_v_1.3 mRNA in Jurkat cells by both shRNAs (1 and 2) resulted in pronounced cell death in comparison to cells treated with nonspecific shRNA (Sh-scr) (Fig. 1d). Taken together, pharmacological inhibition using memantine or genetic inactivation of K_v_1.3 channels impairs survival of Jurkat acute lymphoblastic leukemia cells.

### Memantine potentiates AraC-induced cell death of acute lymphoblastic leukemia cells

**Fig. 2:**
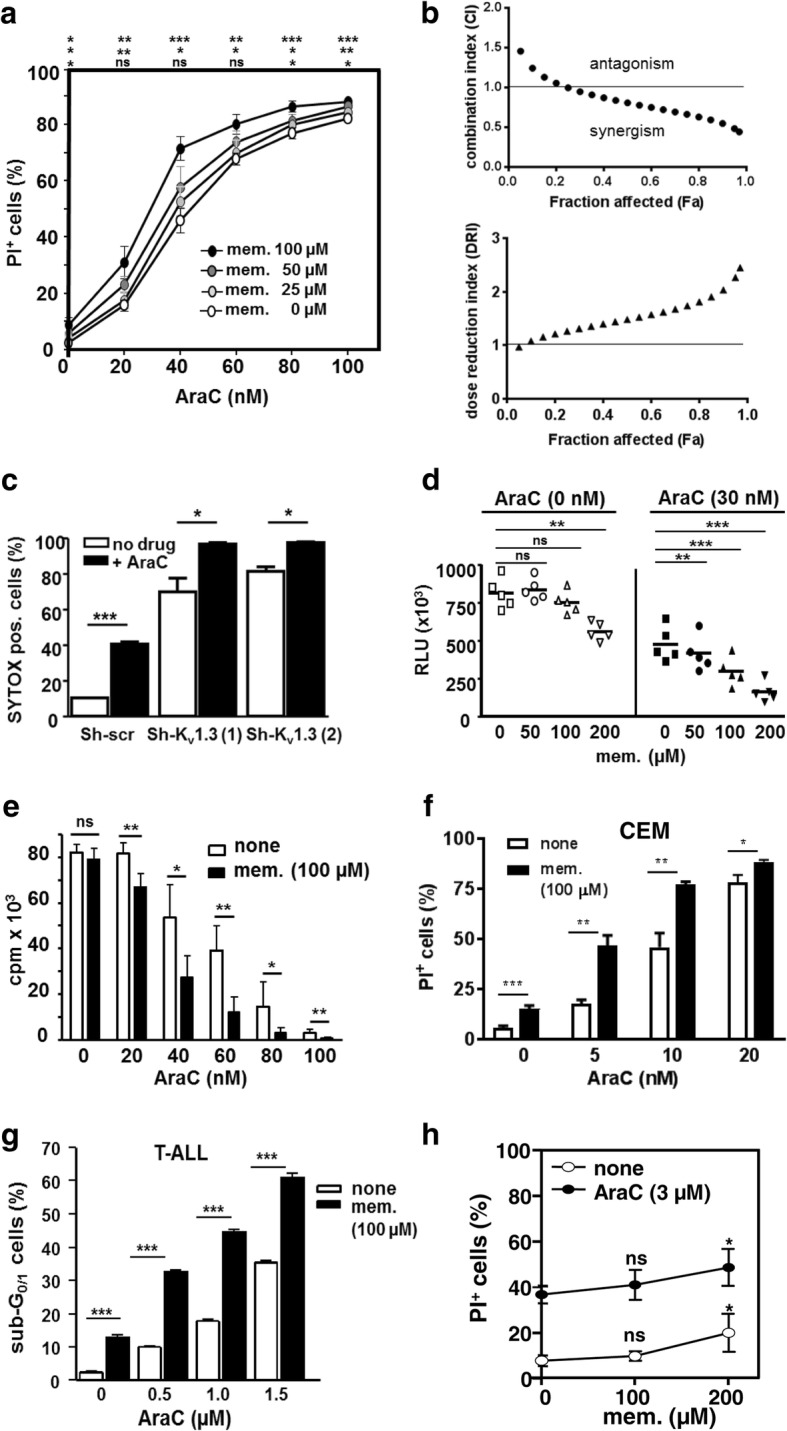
Memantine potentiates AraC-induced cell death and decline of ATP content and cell proliferation. **a** Jurkat cells were cultured with AraC±memantine for 72 h and cell death was determined with PI staining and flow cytometry. The mean ± SD percentage of PI^+^ cells was calculated from *n* = 4 independent experiments. **b** Jurkat cells were cultured with AraC and memantine in constant drug ratios for 72 h. The combination index (CI) for AraC+memantine treatment and the dose reduction index (DRI) for AraC was calculated from *n* = 5 independent experiments with the Chou-Talalay method. **c** Jurkat cells, cultured in duplicates, were infected with lentivirus harboring shRNA against K_v_1.3 channels (Sh-K_v_1.3 (1) and Sh-K_v_1.3 (2)) or scrambled sequence (Sh-scr). 48 h post lentiviral infection, part of Jurkat cells were treated with 20 nM AraC. SYTOX staining was performed at day 5. The mean + SD percentage of SYTOX^+^ cells from duplicate cultures is shown for one out of two experiments. **d** Jurkat cells were cultured without drug, with memantine and AraC±memantine for 72 h and intracellular ATP content was determined with CellTiter-Glo® luminescent assay. Data shows relative light units (RLU) and mean of *n* = 5 independent experiments. **e** DNA synthesis of Jurkat cells treated with AraC±memantine was determined by ^3^[H]-Thymidine incorporation at 72 h; data show mean + SD cpm values of triplicates of one representative experiment (out of 5). **f** CEM cells were cultured in triplicates with the indicated concentrations of AraC±memantine (100 μM) for 72 h. PI staining was used to determine cell death. The graph shows the mean + SD percentage of PI^+^ cells. Data is representative for *n* = 3 independent experiments. **g** PBMC isolated from a newly diagnosed T-ALL patient (78 years of age, 83% blasts) were cultured in triplicates for 72 h with the indicated concentrations of AraC±memantine. The percentage + SD of sub-G_0/1_ cells (indicative of dead cells) was determined with PI staining and flow cytometry. **h** CD3^+^ T cells were isolated from healthy donors and cultured in triplicates with AraC (3 μM) ± memantine for 72 h. Data show the percentage ± SD of PI^+^ cells calculated from *n* = 3 donors. Significance in a-h was determined with Student’s *t*-test with P* < 0.05, P** < 0.01, P*** < 0.001, and ns = not significant

To evaluate whether pharmacological inhibition of K_v_1.3 channels on acute leukemia cells could be a valuable approach to enhance chemotherapeutic efficacy, we assessed the effect of K_v_1.3 channel blockade by memantine in combination with AraC (one of the frontline chemotherapeutic drugs in acute myeloid leukemia treatment). Memantine increased AraC-induced cell death of Jurkat cells in a dose-dependent manner (Fig. 2a). For evaluation of the nature of drug interaction, we used fixed drug ratio design and the Chou-Talalay method [46]. Combination index (CI) values < 1, =1, > 1 indicate synergistic, additive and antagonistic effects. CI values for memantine ranged from 0.99 at the affected fraction (Fa) of 0.25 to 0.44 at Fa 0.97 (97% dead cells) indicating varied effects ranging from additive to synergistic (Fig. 2b). The dose reduction index (DRI) at the Fa of 0.97 was 2.4 (Fig. 2b), i.e. memantine co-treatment allows a 2.4-fold dose reduction of AraC to kill 97% of cells. To complement this data, Jurkat cells with K_v_1.3 channel knockdown were simultaneously treated with AraC. Genetic inactivation of K_v_1.3 channels induced 70–80% cell death (on day 5). Interestingly, addition of AraC (20 nM) further augmented cell death to 95% (Fig. 2c), supporting a cooperative action of AraC and K_v_1.3 channel inhibition in inducing cell death. AraC/memantine co-treatment of Jurkat cells also induced a decline in intracellular ATP level and DNA synthesis compared to cells treated with AraC alone (Fig. 2d and e). To exclude the possibility of a Jurkat cell-specific effect, we analysed AraC-induced cell death of the human acute lymphoblastic leukemia cell line CEM. As in Jurkat cells, K_v_ current in CEM cells is exclusively mediated by K_v_1.3 channels [50]. Again, AraC/memantine co-treatment enhanced cell death of CEM cells compared to cells treated with AraC alone (Fig. 2f). Further, we examined the effect of memantine co-treatment on primary ALL blasts. PBMC from a newly diagnosed T-ALL patient responded to memantine co-treatment with a 2–3-fold increase in the percentage of cells in sub-G_0/1_-phase of cell cycle, representing dead cells, compared to AraC application alone (Fig. 2g). In contrast, memantine co-treatment of peripheral blood T cells of healthy donors increased cell death in combination with AraC only at concentrations beyond 100 μM (Fig. 2h), indicating that primary human T cells are less sensitive to memantine co-treatment than acute leukemic T cells. Hence, pharmacological inhibition of K_v_1.3 channels by memantine potentiates AraC-induced proliferative arrest and cell death in acute lymphoid leukemia cells.

### Pharmacological blockade of K_v_1.3 channels by memantine fosters inhibition of AKT, ERK1/2 and MYC

**Fig. 3:**
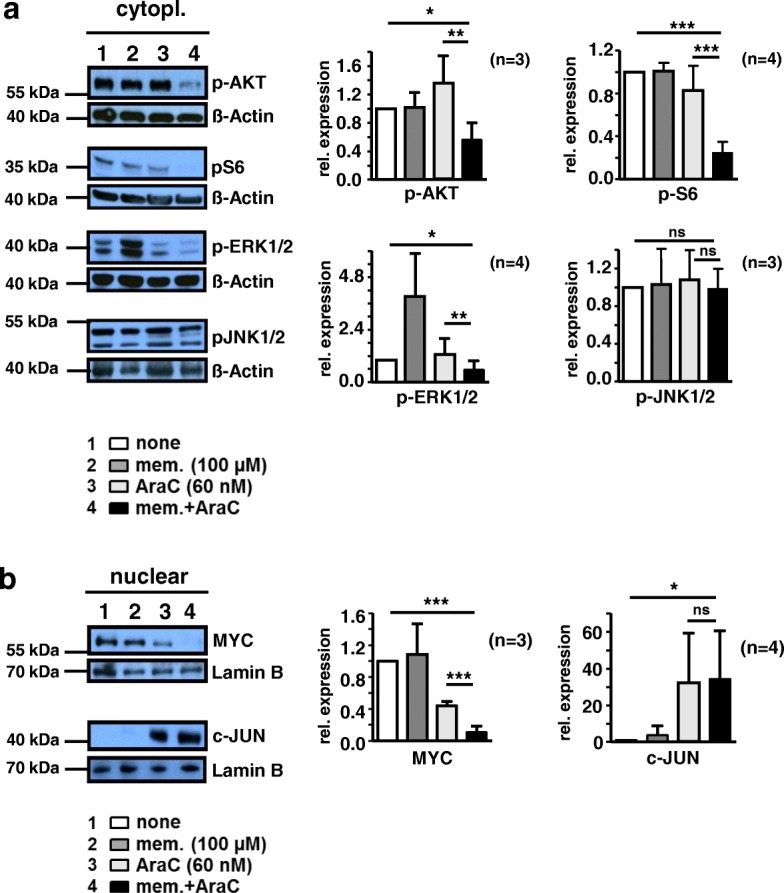
Memantine co-treatment augments inhibition of AKT, ERK1/2 and MYC signaling. **a.** and **b.** Jurkat cells were cultured without drug, with 100 μM memantine, 60 nM AraC, and memantine+AraC for 72 h (lanes 1–4, respectively). **a** Cytoplasmic and **b** nuclear protein samples were immunoblotted for expression of the indicated proteins; ß-Actin and Lamin B expression served as control for protein loading. A representative Western blot is shown for each indicated protein. The data in bar graphs provide the mean + SD relative expression of the indicated protein calculated from densitometric quantifications of *n* = 3–4 independent experiments for each indicated protein

Hyper-activation of signaling cascades, including AKT and ERK1/2, promotes the development of leukemia. Therefore, simultaneous targeting of these signaling cascades might enhance chemotherapeutic efficacy [51, 52]. To delineate molecular events affected by memantine, AraC alone or in combination, we analyzed the activation of AKT, ribosomal component S6, ERK1/2, and JNK1/2 in Jurkat cells. We also monitored nuclear accumulation of MYC, a target of ERK1/2 and AKT signaling (Fig. 3). While co-application of memantine and AraC reduced the level of p-AKT and p-S6 to 42 and 27% of untreated controls, respectively, AraC treatment had no major effect. Memantine treatment alone enhanced the expression of p-ERK1/2, however, concomitant drug treatment reduced p-ERK1/2 by 54% compared to AraC monotherapy. Neither AraC nor AraC/memantine treatment significantly altered the expression of p-JNK1/2 (Fig. 3a), indicating a selective inhibition of signaling molecules by AraC and AraC/memantine. Furthermore, AraC treatment resulted in decreased nuclear MYC and this effect was even stronger upon memantine co-treatment (a further 4-fold decrease) (Fig. 3b). In contrast, AraC and AraC/memantine co-treatment showed a similar nuclear accumulation of the transcription factor JUN, suggesting that JUN induced by AraC is not significantly affected by memantine (Fig. 3b). Thus, in combination with AraC, memantine fosters inhibition of AKT, ERK1/2 and MYC signaling in Jurkat cells, key regulators of proliferation and survival in acute leukemia.

### Memantine enhances CytC release and Caspase-9 activation

**Fig. 4:**
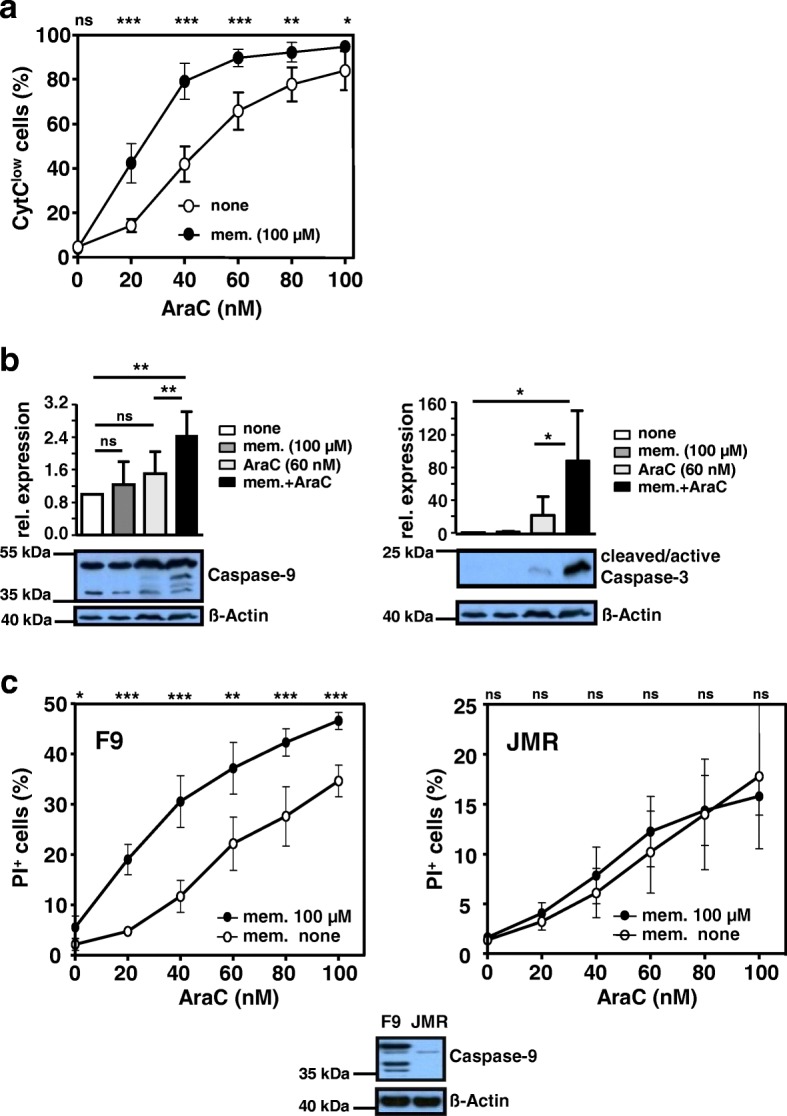
Memantine co-treatment results in increased CytC release and activation of Caspase-9 and Caspase-3. **a** Jurkat cells were cultured with memantine and AraC±memantine as indicated. After 72 h intracellular CytC level was determined with flow cytometry. CytC^low^ cells represent apoptotic cells having released mitochondrial CytC. Data of *n* = 5 independent experiments is shown as mean ± SD. **b** Expression of Caspase-9 and the cleaved active form of Caspase-3 in Jurkat cells treated without and with drug as indicated was analyzed by Western blot. The data in bar graphs give the mean + SD relative expression of each indicated protein calculated from densitometric quantifications of *n* = 4 independent experiments. **c** Cell death of F9 and JMR cells cultured without or with AraC±memantine for 72 h was determined with PI staining. The percentage of PI^+^ cells of *n* = 5 independent experiments is shown as mean ± SD. Western blot shows Caspase-9 and ß-Actin expression in F9 and JMR cells

Since memantine potentiated AraC-induced cell death, we investigated distinct apoptosis mechanisms. Analyzing the extrinsic apoptotic pathway, Caspase-8 levels in AraC- and AraC/memantine-treated Jurkat cells were similar (Additional file 1: Figure S2a) consistent with a previous report on AraC-induced apoptosis [53]. In addition, in Caspase-8-deficient (C8) and parental (A3) Jurkat cells memantine co-treatment led to a similar enhancement of AraC-induced cell death (Additional file 1: Figure S2b), indicating that the effects of memantine are independent of Caspase-8 activity. We next studied whether memantine acts on the intrinsic apoptotic pathway. Memantine co-treatment augmented the percentage of CytC^low^ Jurkat cells, representing apoptotic cells having released mitochondrial CytC, by 10–40% (Fig. 4a). Furthermore, expression of Caspase-9 fragments, which are indicative of active Caspase-9 induced by mitochondrial CytC release, was elevated in Jurkat cells upon co-treatment in comparison to AraC monotherapy (Fig. 4b). Combined drug treatment also resulted in a 4-fold increase in the expression of the cleaved active form of Caspase-3 (Fig. 4b). The important contribution of Caspase-9 activation for cell death potentiated by memantine was supported by analysis of Caspase-9-deficient (JMR) and Caspase 9-reconstituted (F9) cells. Memantine significantly enhanced AraC-induced cell death in F9, but not in JMR cells, which were less sensitive to AraC treatment than F9 cells (Fig. 4c). Overall, the results allow the conclusion that memantine enhances AraC-induced cell death by promoting CytC release and activation of Caspase-9 and Caspase-3, thus amplifying intrinsic apoptosis.

### Memantine potentiates AraC cytotoxicity in AML cell lines and primary AML blasts

**Fig. 5:**
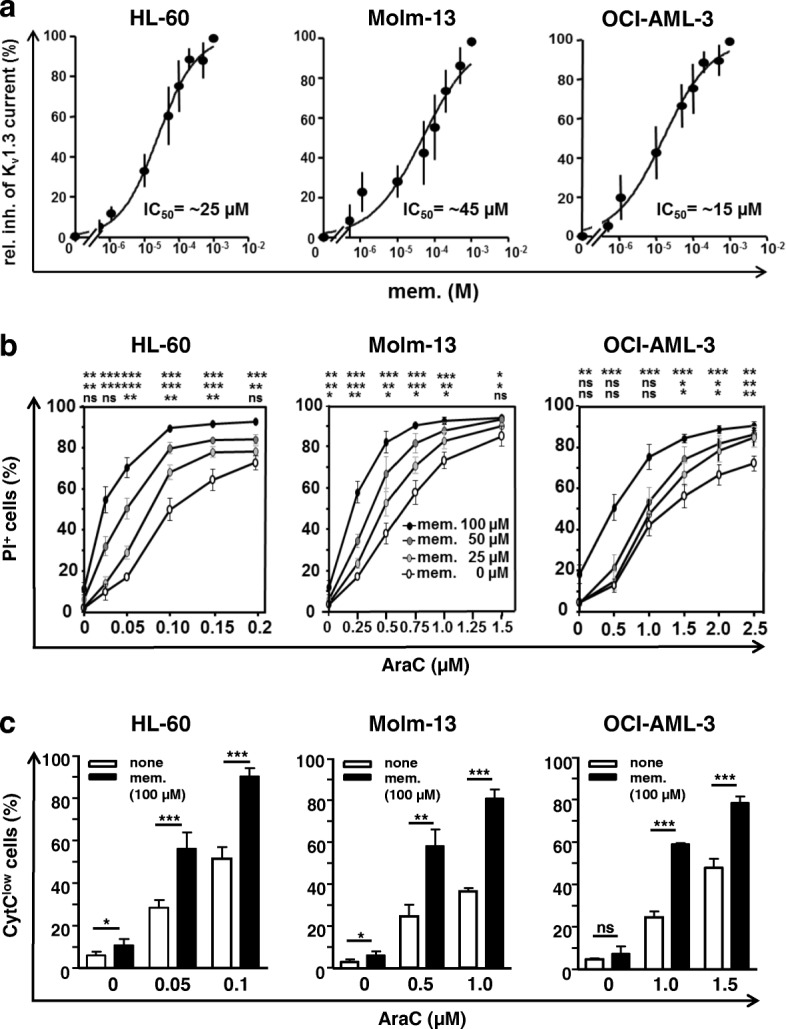
Memantine potentiates AraC-induced cell death of AML cell lines. **a** Dose response relationship for memantine is shown for isolated K_v_1.3 currents recorded from the acute myeloid leukemia cell lines HL-60, Molm-13 and OCI-AML-3. Each data point represents the mean ± SD of 5–7 cells from *n* = 3 experiments. **b** HL-60 and Molm-13 cells were treated with AraC±memantine for 3 days and OCI-AML3 cells for 5 days. Cell death was determined with PI staining and flow cytometry. For each cell line the percentage ± SD of PI^+^ cells was calculated from *n* = 4 independent experiments. **c** Graphs show the percentage + SD of CytC^low^ AML cells at day 3 (day 5 for OCI-AML-3 cells); for each cell line data was calculated from *n* = 4 independent experiments

Since in adults acute leukemia mainly comprises myeloid differentiation, we asked whether AraC/memantine co-treatment could be an option in therapy of AML. Therefore, we analyzed the human myeloid leukemia cell lines HL-60, Molm-13 and OCI-AML-3. Surface expression of K_v_1.3 channels on these AML cell lines was verified by flow cytometry (Additional file 1: Figure S3a) [54]. Patch-clamp studies confirmed their functionality and the average expression number could be calculated to 505, 431 and 464 K_v_1.3 channels per cell for HL-60, Molm-13 and OCI-AML-3, respectively (Additional file 1: Figure S3b). Memantine blocked K_v_1.3 channel activity with IC_50_ values of 25, 45 and 15 μM for HL-60, Molm-13 and OCI-AML-3 cells (Fig. 5a). In combination with AraC, 25–100 μM memantine further enhanced AraC-induced cell death in the AML cell lines investigated (Fig. 5b). CI values calculated from constant drug ratio experiments showed synergistic effects with CI values at Fa 0.97 of 0.46, 0.76 and 0.71 for HL-60, Molm-13 and OCI-AML-3 cells, respectively, and DRI values at Fa 0.97 of 7.7, 1.8 and 5.4 (Additional file 1: Figure S3c). Co-application of memantine also significantly raised the percentage of CytC^low^ cells in each AraC-treated AML cell line (Fig. 5c), indicating enhanced mitochondrial dysfunction. Immunoblotting of Molm-13 cells revealed that combined AraC/memantine treatment enhances the decline of p-AKT and p-ERK1/2 compared to AraC application alone (Additional file 1: Figure S3d). Altogether, these results suggest that memantine potentiates AraC cytotoxicity through similar mechanisms in acute lymphoid and myeloid leukemia cell lines.

**Fig. 6:**
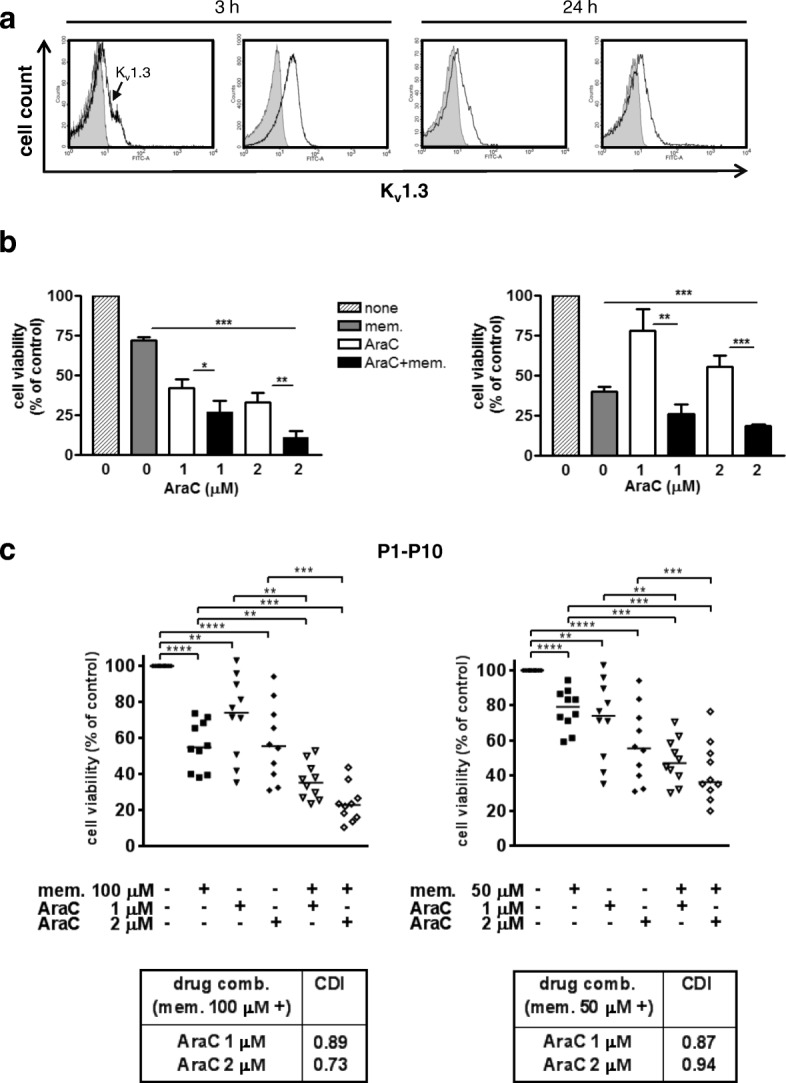
Memantine potentiates cell death of patients’ myeloid leukemic blasts. **a** Bone marrow (BM) cells of AML patients were analysed for surface expression of K_v_1.3 by flow cytometry. Histograms show K_v_1.3 profiles of viable CD117^+^ leukemic blasts cultured for 3 or 24 h and are representative for eight BM samples. Cells without K_v_1.3 staining are depicted in grey histograms. **b.** and **c.** BM cells of 10 AML patients (Additional file 1: Table S1) were cultured without any drug, with memantine alone (100 μM lower left graph, 50 μM lower right graph), with 1 μM and 2 μM AraC alone, and with AraC plus memantine for 48 h. Cell death was determined with SYTOX/Annexin-V staining and flow cytometry. SYTOX^−^Annexin-V^−^ cells were considered as viable cells. For each patient, cell viability of untreated (control) AML cells was set as 100% and cell viability of treated AML cells was related to the corresponding control sample. **b** The graphs show data of two representative patients (cells were treated with 100 μM memantine). **c** Graphs provide the relative cell viability of BM cells of 10 AML patients. Coefficient of drug interaction (CDI) values for combined memantine and AraC treatments are provided in the boxes; CDI < 1 indicates synergism and CD > 1 antagonism

To validate memantine’s therapeutic potential in AML, we analyzed leukemic blasts from bone marrow of 10 AML patients (Additional file 1: Table S1). Low surface expression of K_v_1.3 was detectable on all analysed viable CD117^+^ AML blasts (Fig. 6a). Although sensitivity of AML cells towards AraC and memantine varied between individuals, co-treatment with memantine (100 and 50 μM) further increased cell death of AraC-treated AML samples (Fig. 6b and c). Nature of memantine and AraC interaction was evaluated by calculating coefficient of drug interaction (CDI) values. Interestingly, CDI values were less than one indicating synergism when the cells were simultaneously treated with memantine and AraC. Compared to cell lines, patients’ primary leukemic cells were more sensitive to memantine monotherapy as it induced pronounced cell death in the absence of AraC. The results from primary acute leukemic blasts suggest that combined AraC/memantine treatment could be a therapeutic strategy to enhance cell death in acute leukemia.

## Discussion

In line with reports underscoring the importance of K_v_1.3 channels in survival of leukemic B cells [28, 55] and various cancer cell lines [54], our data provide initial evidence that K_v_1.3 channels are druggable targets in chemotherapy of T-ALL and AML. To address the clinical need for a pharmacological K_v_1.3 channel inhibitor, we used memantine, which shows an excellent safety profile and is licensed for NMDAR antagonism in advanced Alzheimer disease [35]. Memantine inhibited K_v_1.3 channels and potentiated AraC-induced cell death of acute lymphoid (Jurkat, CEM) and myeloid (HL-60, Molm-13, OCI-AML-3) leukemia cell lines (representing highly chemoresistant aggressive clones) as well as primary acute leukemic blasts. Synergistic drug effects were observed for the analyzed cell lines and AML blasts. Jurkat and Molm-13 cells showed enhanced reduction of ERK1/2 and AKT signaling upon drug co-treatment and enhanced cell death of Jurkat and AML cell lines was connected with increased CytC release. Since mitochondrial K_v_1.3 channels contribute to the intrinsic apoptotic pathway upon a pre-existing apoptotic stimulus [23, 25], AraC may act as a sensitizer for inhibition of K_v_1.3 channels. Thus, addition of memantine to AraC results in enhanced CytC release and activation of Caspase-9 and Caspase-3. In primary ALL and AML blasts memantine monotherapy already induced cell death, but combination of memantine and AraC was most effective in cell death induction of leukemic blasts. In contrast to acute leukemic cells, primary non-malignant T lymphocytes did not show a relevant potentiation of AraC by memantine. This may relate to the enhanced susceptibility of malignant cells to reactive oxygen species produced at the mitochondria upon apoptosis induction [23, 25].

Using murine lymphocytes, we previously reported that memantine blocks K_v_1.3 and K_Ca_3.1 channels [37]. Therefore, we do not exclude a possibility of memantine affecting K_Ca_3.1 channels (and maybe other ion channels) [50, 54, 56, 57] which might contribute to memantine’s potentiation of AraC cytotoxicity. Compared to memantine treatment alone, knockdown of K_v_1.3 mRNA in Jurkat cells was more effective in inducing cell death. This could in part be due to transient pharmacological blockade of cell surface K_v_1.3 channels by memantine and a compensatory up-regulation of K_v_1.3 channel expression and activity as a mechanism of drug resistance [39, 54].

The PI3K-AKT-mTOR and ERK1/2 signaling pathways are commonly activated in acute leukemia and confer poor prognosis [52]. PI3K-AKT-mTOR inhibitors have been used in clinical trials [13, 58], however, the induction of feedback and compensatory mechanisms, like aberrant ERK1/2 activation leading to phosphorylation of ribosomal protein S6, may have contributed to drug resistance [6]. Further, treatment of T-ALL cell lines with PI3K and mTOR inhibitors led to an up-regulation of MYC [59, 60]. Here we found that memantine co-treatment targets several central signaling cascades as it concurrently decreased AKT, ERK1/2 and MYC signaling while increasing mitochondrial CytC release and Caspase-9/Caspase-3 activation. Since memantine/AraC co-treatment led to effective killing of lymphoid and myeloid leukemia cell lines and patients’ leukemic blasts with different genetic background, memantine may act through ‘common’ mechanisms that feed into or extend the AraC-induced cell death machinery. Beside inhibition of AKT-mTOR and ERK1/2 signaling, these may include inhibition of calcium signaling and cell cycle by altering the membrane potential [49] and increase of intracellular potassium concentrations, which inhibited AKT-mTOR activity in T-effector cells by unknown mechanisms [61].

Memantine is an orally given drug with excellent bioavailability and long half-life. Steady-state memantine concentrations in serum of treated (10–20 mg/day) Alzheimer patients were reported to be < 1.0 μM [35, 36]. In our study, effective memantine concentrations (given only once at begin of cell culture) ranged from 25 to 100 μM. However, daily therapeutic doses of memantine block K_v_1.3 channels on blood T cells and inhibit T cell function in vivo*,* whereas inhibition of human T cell function in vitro required higher memantine concentrations [39]. Various pharmacologic factors such as drug metabolites, half-life, daily dosing, and niche specific drug-cell interactions might account for the difference of in vitro versus in vivo drug effectiveness. Memantine is being tested in several disease settings without showing severe side effects even in elderly patients and at higher drug doses. As a licensed drug proven to inhibit K_v_1.3 channels in vivo, memantine seems to be suited for testing a potential cooperative action in AraC therapy of acute leukemia.

## Conclusion

Our data support the concept of targeting K_v_1.3 channels in ALL and AML therapy and, though in vivo studies remain to be performed, suggest memantine as a potential intensifier of AraC-based treatments of different subtypes of acute leukemia, particular in palliative low-dose AraC monotherapy of patients.

## Additional files

Additional file 1: **Table S1.** Characteristics of AML patients. **Figure S1.** a K_v_1.3 expression on Jurkat cells; grey histogram shows isotype staining. b Knockdown of K_v_1.3 mRNA in Jurkat cells via lentivirus harboring Sh-K_v_1.3 (1), Sh-K_v_1.3 (2) or scrambled (Sh-scr) sequence. Data give the relative mean + SEM expression of K_v_1.3 mRNA from triplicate cultures of one experiment at day 3, *n* = 6. c K_v_1.3 expression on CEM cells; grey histogram shows unstained cells. **Figure S2.** a Jurkat cells were cultured without drug, 100 μM memantine, 60 nM AraC, and memantine+AraC for 72 h. Caspase-8 and β-actin  expression was analysed by Western blot. Data show mean + SD relative expression of Caspase-8; *n* = 4. b Parental A3 and Caspase-8-deficient C8 cells were treated with AraC±memantine for 72 h; mean ± SD percentage of PI^+^ cells was calculated from *n* = 5. Western blot shows Caspase-8 and β-actin expression. Student´s *t*-test: P** < 0.01, P*** < 0.001, ns = not significant. **Figure S3.** a K_v_1.3 expression on HL-60, Molm-13, OCI-AML-3; grey histograms show unstained cells. b Number of K_v_1.3 channels/cell of HL-60, Molm-13, and OCI-AML-3. Data show mean ± SEM K_v_1.3 channel number of *n* = 4-5 experiments for each cell line and mean K_v_1.3 number of all cells. c HL-60, Molm-13, OCI-AML-3 cells were cultured with AraC and memantine at fixed drug ratios for 72 h; percentage of PI^+^ cells was determined. For each cell line, combination index (CI) and dose reduction index (DRI) for AraC were calculated from *n* = 4-5 using Chou-Talalay method. CI < 1 drug synergism, CI = 1 additivity, CI > 1 drug antagonism. d Molm-13 cells were cultured without drug, 100 μM memantine, 250 nM AraC, and memantine+AraC for 46 h. Cytoplasmic expression of indicated proteins was analysed by Western blot; *n* = 2-3. (PDF 226 kb)

## Abbreviations

AraC: cytarabine
CI: combination index
CytC: cytochrome c
DRI: dose reduction index
Mem: memantine
PI: propidium iodide
