# Supplementary material for: Memantine potentiates cytarabine-induced cell death of acute leukemia correlating with inhibition of Kv1.3 potassium channels, AKT and ERK1/2 signaling
Source: Cell Commun Signal. 2019 Jan 16;17:5. doi: 10.1186/s12964-018-0317-z (PMC6335768; doi:10.1186/s12964-018-0317-z)
Supplement: Supplementary file 1 — Table S1. Characteristics of AML patients. Figure S1. a Kv1.3 expression on Jurkat cells; grey histogram shows isotype staining. b Knockdown of Kv1.3 mRNA in Jurkat cells via lentivirus harboring Sh-Kv1.3 (1), Sh-Kv1.3 (2) or scrambled (Sh-scr) sequence. Data give the relative mean + SEM expression of Kv1.3 mRNA from triplicate cultures of one experiment at day 3, n = 6. c Kv1.3 expression on CEM cells; grey histogram shows unstained cells. Figure S2. a Jurkat cells were cultured without drug, 100 μM memantine, 60 nM AraC, and memantine+AraC for 72 h. Caspase-8 and β-actin expression was analysed by Western blot. Data show mean + SD relative expression of Caspase-8; n = 4. b Parental A3 and Caspase-8-deficient C8 cells were treated with AraC±memantine for 72 h; mean ± SD percentage of PI+ cells was calculated from n = 5. Western blot shows Caspase-8 and β-actin expression. Student´s t-test: P** < 0.01, P*** < 0.001, ns = not significant. Figure S3. a Kv1.3 expression on HL-60, Molm-13, OCI-AML-3; grey histograms show unstained cells. b Number of Kv1.3 channels/cell of HL-60, Molm-13, and OCI-AML-3. Data show mean ± SEM Kv1.3 channel number of n = 4-5 experiments for each cell line and mean Kv1.3 number of all cells. c HL-60, Molm-13, OCI-AML-3 cells were cultured with AraC and memantine at fixed drug ratios for 72 h; percentage of PI+ cells was determined. For each cell line, combination index (CI) and dose reduction index (DRI) for AraC were calculated from n = 4-5 using Chou-Talalay method. CI < 1 drug synergism, CI = 1 additivity, CI > 1 drug antagonism. d Molm-13 cells were cultured without drug, 100 μM memantine, 250 nM AraC, and memantine+AraC for 46 h. Cytoplasmic expression of indicated proteins was analysed by Western blot; n = 2-3. (PDF 226 kb) [file 12964_2018_317_MOESM1_ESM.pdf]

Table S1: Characteristics of AML patients.

Patient code	Age	FLT3-ITD status	NPM1 status	Percentage of blasts (BM)
P1	74	neg.	neg.	64
P2	68	neg.	pos.	n.d.
P3	42	pos.	pos.	67
P4	47	neg. (TKD+)	neg.	36
P5	29	neg.	neg.	20
P6	74	neg.	n.d.	80
P7	60	pos.	pos.	91
P8	69	n.d.	n.d.	> 90
P9	62	neg. (TKD+)	n.d.	43
P10	n.d.	n.d.	n.d.	46

FLT3-ITD: fms-like tyrosine kinase 3-internal tandem duplication; NPM1: nucleophosmin 1; BM: bone marrow; neg.: negative, pos.: positive, TKD+: tyrosine kinase domain mutated, n.d.: not determined

Figure S1

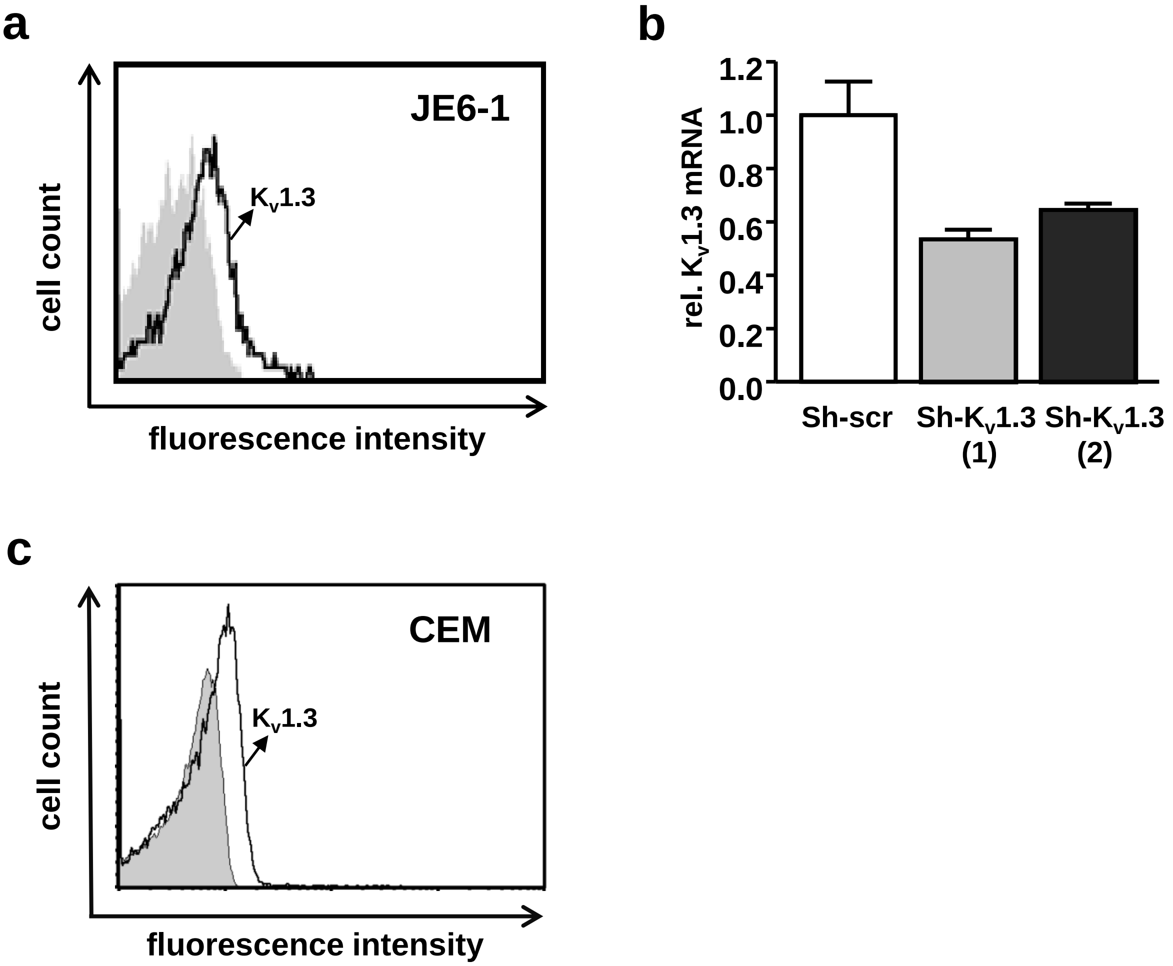
a
JE6-1
K_v1.3
cell count
fluorescence intensity
b
rel. K_v1.3 mRNA
1.2
1.0
0.8
0.6
0.4
0.2
0.0
Sh-scr
Sh-K_v1.3 (1)
Sh-K_v1.3 (2)
c
CEM
K_v1.3
cell count
fluorescence intensity

Figure S2

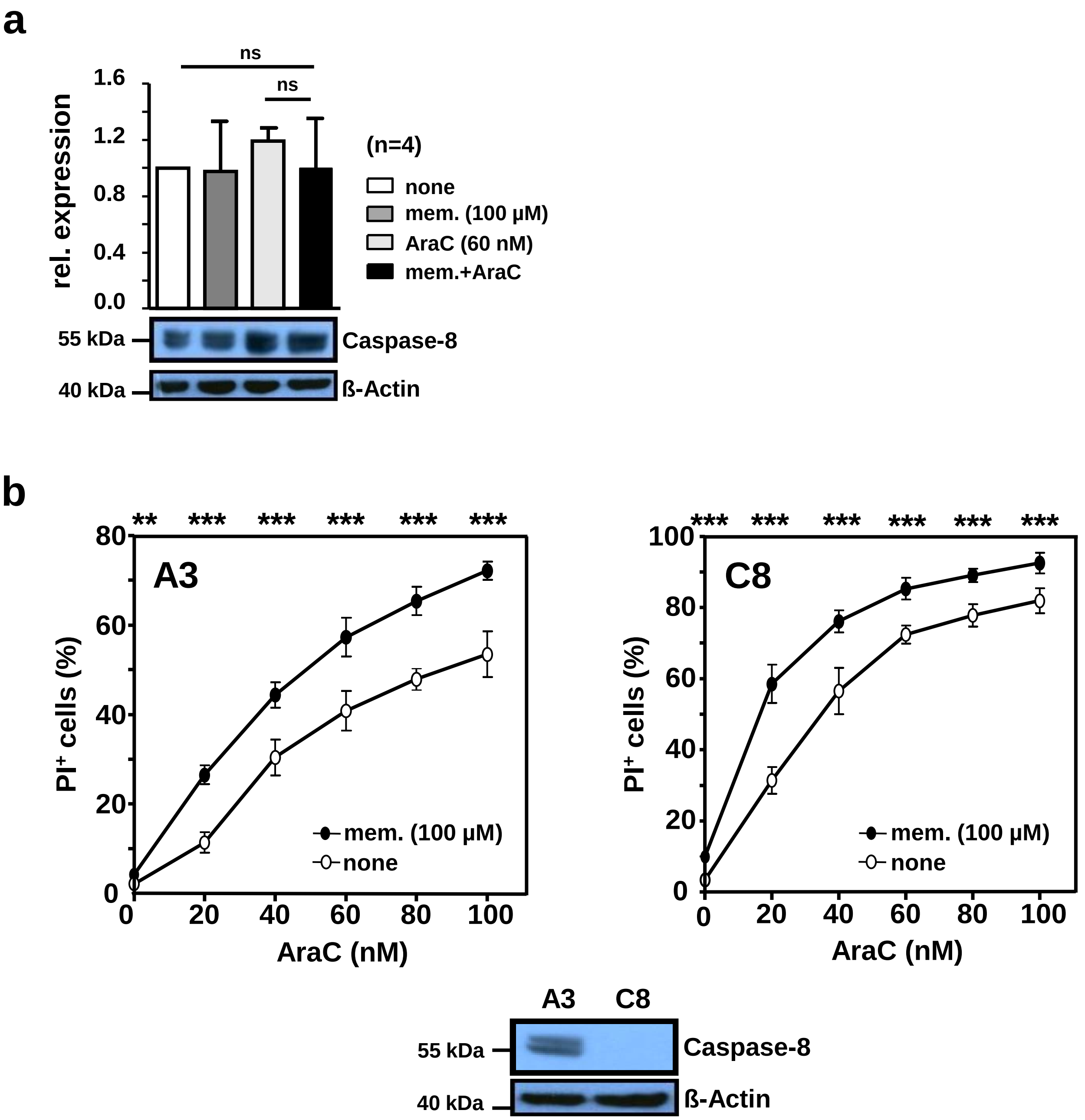

a
ns
ns
1.6
1.2
0.8
0.4
0.0
rel. expression
(n=4)
none
mem. (100 µM)
AraC (60 nM)
mem.+AraC
55 kDa
Caspase-8
40 kDa
ß-Actin
b
** *** *** *** *** ***
A3
PI⁺ cells (%)
0
20
40
60
80
100
AraC (nM)
mem. (100 µM)
none
*** *** *** *** *** ***
C8
A3
C8
55 kDa
Caspase-8
40 kDa
ß-Actin

Figure S3

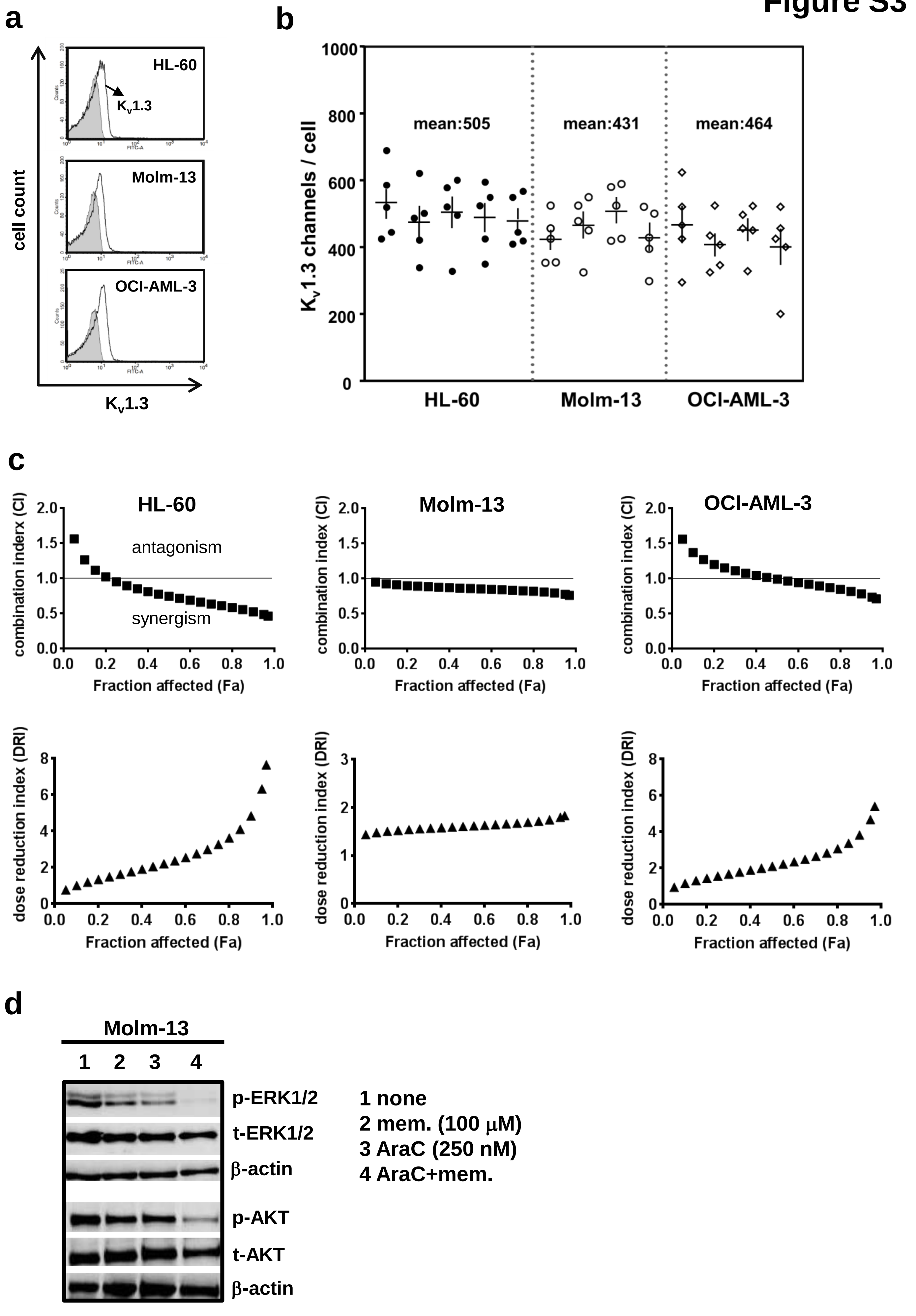
a
b
c
d
HL-60
Molm-13
OCI-AML-3
cell count
K_v1.3
K_v1.3 channels / cell
mean:505
mean:431
mean:464
combination index (CI)
antagonism
synergism
Fraction affected (Fa)
dose reduction index (DRI)
p-ERK1/2
t-ERK1/2
β-actin
p-AKT
t-AKT
β-actin
1 none
2 mem. (100 μM)
3 AraC (250 nM)
4 AraC+mem.
